# Correlating the site of tympanic membrane perforation with Hearing loss

**DOI:** 10.1186/1472-6815-9-1

**Published:** 2009-01-04

**Authors:** Titus S Ibekwe, Onyekwere G Nwaorgu, Taiwo G Ijaduola

**Affiliations:** 1 ENT Division, Irrua Specialist Teaching Hospital and College of Medicine, Ambrose Alli University, Ekpoma, Nigeria; 2 Department of Otorhinolaryngology, University College Hospital Ibadan and College of Medicine University of Ibadan, Ibadan, Nigeria

## Abstract

**Background:**

It is recognized that the size of tympanic membrane(TM) perforation is proportional to the magnitude of hearing loss, however, there is no clear consensus on the effect of the location (site) of the perforation on the hearing loss. Hence the study is set to investigate the relationship between the location of perforation on TM and hearing loss.

**Methods:**

A cross-sectional prospective study of consecutive adult patients with perforated TM conducted in the ENT clinic of University College Hospital Ibadan between January 1st 2005 and July 31st 2006. Instruments used for data collection/processing include questionnaires, video and micro-otoscopy, Pure tone audiometer, image J and SPSS packages.

**Results:**

Sixty-two patients (22-males, 40-females), aged 16–75 years (mean = 35.4 +/- 4) with 77 perforated ear drums were studied and 15(24.2%) had bilateral TM perforations, 21 (33.9%) right unilateral and 26(41.9%) left unilateral. The locations of the TM perforations were 60(77.9%) central, 6(9.6%) antero-inferior, 4(5.2%) postero-inferior, 4(5.2%) antero-superior and 3(3.9%) postero-superior respectively with sizes ranging from 1.51%–89.05%, and corresponding hearing levels 30 dB – 80 dB (59% conductive and 41% mixed). Fifty-nine percent had pure conductive hearing loss and the rest mixed. Hearing losses (dBHL) increased with the size of perforations (P = 0.01, r = 0.05). Correlation of location of perforations with magnitude of hearing loss in acute TM perorations was (P = 0.244, r = 0.273) and for chronic perforations (p = 0.047 & r = 0.31).

**Conclusion:**

The location of perforation on the tympanic membrane (TM) has no effect on the magnitude of hearing loss in acute TM perforations while it is significant in chronic ones.

## Background

Apart from conduction of sound waves across the middle ear, the tympanic membrane, also sub-serves a protective function to the middle ear cleft and round window niche. Intact tympanic membrane protects the middle ear cleft from infections and shields the round window from direct sound waves which is referred to as 'round window baffle'.[1] This shield is necessary to create a phase differential so that the sound wave does not impact on the oval and round windows simultaneously. This would dampen the flow of sound energy being transmitted in a unilateral direction from the oval window through the perilymph. It has been found that the effect of the enhanced ratio of the surface area of the tympanic membrane to that of the oval window increases the sound pressure by about 27 decibel (dB) whereas the lever action of ossicles contributes about 3 decibel (dB). [2,3]

A perforation on the tympanic membrane reduces the surface area of the membrane available for sound pressure transmission and allows sound to pass directly into the middle ear. As a result, the pressure gradient between the 'inner' and 'outer' surfaces of the membrane virtually becomes insignificant. The effectiveness with which the tympanic membrane transmits motion to the ossicular chain is thus impaired along with the level of hearing.[4] It has been established that the larger the perforation on the tympanic membrane, the greater the decibel loss in sound perception. A total absence of the tympanic membrane would lead to a loss in the transformer action of the middle ear.[5] The location of the perforation is believed by some schools of thought to have a significant effect on the magnitude of hearing loss.[6] For instance, posterior quadrant perforations are believed to be worse than the anterior ones because of the direct exposure of the round window to sound waves and perforations at or near the site of tympanic membrane attachment to manubrium have more severe effects than those of comparable size at different sites.[6] However, some workers believe that there is no significant effect associated with location of the perforation.[5,7] This divergent opinion, informed undertaking the study, set to investigate the relationship between the location of perforation on TM and the magnitude of conductive hearing loss with a view to contributing to the body of knowledge on this subject.

## Methods

### Study design

This is a cross-sectional prospective study conducted between January 2005 and July 2006 with a target population of consecutive adults (age 15 years and above), seen in the Ear Nose and Throat(ENT) clinic of the University College Hospital (UCH) Ibadan Nigeria, with tympanic membrane perforations.

### Study location

UCH is a tertiary health institution and a major referral center in Nigeria and West African sub-region, located within Ibadan metropolis, population density of about 3.85 million [8] in south west Nigeria.

### Sample size

Sixty two patients with 77 tympanic membrane perforations participated in the study.

### Sampling techniques

Ethical clearance for the study was obtained from the Joint UCH/University of Ibadan Institutional review board. All consecutive adults seen in the ENT clinic within the one year period were screened for tympanic membrane perforations. Consent (Informed, written and well understood) was obtained from each patient with tympanic membrane perforation/s. The aims and objectives of the study, the benefits to be obtained, the confidentiality of participants and results, the voluntary nature of participation and free-will to withdraw from the study without penalty were clearly spelt out to the participants.

### Data collection

Each of the participants was interviewed with a pre tested structured questionnaire (additional file 1) and examined clinically to assess the features of the tympanic membrane perforation. Video otoscopy of all the ears with **Welch-Allyn compact Video Otoscope System **[model 23120 (NTSC) and 23120P (PAL)] were done. All images were adapted through Cute TV USB [Model no-03020701] and recorded on the computer [DELL INSPIRON-600m Pentium M]. Using **Image J **[version 1.35j of Wayne Rasband, National Institutes of Health U.S.A] geometrical package; the area of the perforation (P) and that of the entire tympanic membrane (T) were calculated. Then, the percentage area of perforation (P/T × 100%) for each ear was obtained. For the purpose of this study, the tympanic membrane was comprehensively divided into five segments for clarity (1–4 represent the four quadrants; and 5 involvement of more than one quadrant).

(1) anterosuperior, (2) posterosuperior, (3) anteroinferior, (4) posteroinferior and (5) central for the localization of the site of perforation.

The patients' hearing levels in decibel were assessed with a biologically calibrated ***Kamplex diagnostic audiometer ****at frequencies 250 KH, 500 KH, 1000 KH, 2000 KH, 4000 KH and 8000 KH respectively *in an acoustically treated sound proof boot. Air and bone conduction thresholds were determined. The Mean hearing loss was calculated through the pure tone average taken at 500 Hz, 1000 Hz and 2000 Hz for each site of perforations (1 to 5). All established cases of actively discharging ears and ototoxicity were excluded from the study in order to remove their confounding effects. Despite this, the audiogram of some TM perforations (chronic) gave mixed hearing losses, which is at variance from the expected conductive hearing losses from solely TM perforations. Whereas, the acute TM perforations with purely conductive hearing losses formed a second group. The two groups were analyzed separately and their results compared.

For the purpose of the study acute TM perforations were defined as those TM perforations resulting from trauma and acute otitis media of two weeks duration or less.

### Data processing and analysis

These were carried out with computer software Statistical Package for Social Sciences (version 11; SPSS; Chicago). The sites and sizes of the tympanic membrane perforations were separately correlated with the magnitude of hearing losses through Kruskal-Wallis and Pearson's test. The t-test was applied were appropriate.

### Limitation of the study

This includes the inability to perfectly control all confounding factors especially in chronic tympanic membrane perforations that could have affected the audiometric assessment of the patient since the study was based on live patients and not models.

## Results

Sixty-two patients (22-males, 40-females) with 77 perforated ear drums and age range 16–75 years (mean = 35.4 ± 4) were studied. Bilateral TM perforations were seen in 15 patients (24.2%), right unilateral in 21 patients (33.9%) and left unilateral in 26(41.9%) respectively. For details see 'Additional file 2' below.

The Sites\locations of perforations on the tympanic membrane were correlated with their Mean hearing levels (dB) using the Kruska-Walli's Test (K-W Test). The coefficients of correlation (r) for ears with conductive and mixed Tm perforations were 3.930 and 3.556, while their P-values (P) were 0.313 and 0.046 respectively. See 'Additional files 3, 4 and 5' for the sites of perforations with the Mean hearing losses, and the results of the statistical correlations.

Where, TM-Tympanic membrane, SEM-Standard error of mean K-W test-Kruskal-Walli's testing

The sizes (% perforations = P/T × 100%) of perforation ranged from 1.51%–89.05%, with corresponding hearing levels 30 dB – 80 dB. The TM perforations showed a positive correlation with the magnitude of hearing loss (dBHL) using Pearson's correlational test (p = 0.01, r = 0.05).

## Discussion

The statistical analysis of the locations of Tm perforation in patients with pure conductive hearing loss showed no correlation with the magnitude of hearing losses recorded. This suggests that the position of the TM perforations in acute TM perforations does not affect the resultant magnitude of conductive hearing loss. Rather, this phenomenon is dependent on the size of the perforation and state of other components of the conductive pathway and the middle ear.[9,10] This is in consonance with the reports of Voss et al and other workers whose results showed no effect of position of the tympanic membrane perforation on hearing loss using a model.[5,11,12]

On the contrary, we observed that the location of the perforations correlate positively with magnitude of hearing loss in ears with chronic "complicated" TM perforations resulting in a mixed hearing loss. The posterosuperior segment was most outstanding in this regard. The differentials in the predisposition to superimposition of diseases on the middle ear relative to the location of TM perforation is the most probable explanation, since it is proven that marginal and posterosuperior perforations are more prone, although not exclusively; to such disease like cholesteatoma.[13,14]

This is in agreement with the earlier observations by Ahmad and Ramani.[6] It also conforms to the historic findings of Bekesy[15], Payne and Gither [16], that the position of the TM perforations affects the magnitude of hearing loss. However the effect of direct impaction of sound energy into the middle ear leading to the loss of "round window baffle", as they suggested; may not be the only reason bearing in mind the complex mode of sound transmission across the middle ear as shown by recent models on sound-energy transmission through TM into the middle ear. [17-19] Furthermore, the non conformity of acute TM perforations to this hypothesis strongly suggests other factors [20] beyond effect on sound transmission.

## Conclusion

The location of perforation on the tympanic membrane has no effect on the magnitude of hearing loss in acute tympanic membrane perforations. However, it has a significant impact in chronic tympanic membrane perforations.

## Competing interests

The authors declare that they have no competing interests.

## Authors' contributions

**TSI **was the principal investigator, who conceived the idea of carrying out the project and did the write-up of the work. **OGN **participated actively in the data collection, literature search and proof reading of the manuscript while **TGI **was the supervisor of the project.

## Pre-publication history

The pre-publication history for this paper can be accessed here:



## Supplementary Material

Additional file 1 bmc ent appendix 1.doc is a copy of the questionnaire we used in the study. Click here for file

Additional file 2 bmc ent table 1.docx outlines the Frequency of Sites (locations) of tympanic membrane perforations. Click here for file

Additional file 3 bmc ent table 2a.docx illustrates the Statistical correlation of sites of perforations with hearing loss in acute TM perforations. Click here for file

Additional file 4 bmc ent table 2b.doc shows Statistical correlation of sites of perforations with hearing loss in chronic TM perforations. Click here for file

Additional file 5 bmc ent table 2c.doc outlines Statistical correlation of sites of perforations with hearing loss in chronic TM perforations (mixed hearing loss) with elimination of the sensorineural component (A-B gap). Click here for file
