# Supplementary material for: Correlating the site of tympanic membrane perforation with Hearing loss
Source: BMC Ear Nose Throat Disord. 2009 Jan 4;9:1. doi: 10.1186/1472-6815-9-1 (PMC2631525; doi:10.1186/1472-6815-9-1)
Supplement: Additional file 1 — bmc ent appendix 1.doc is a copy of the questionnaire we used in the study. [file 1472-6815-9-1-S1.doc]

**APPENDIX I**

**QUESTIONNAIRE**

QUESTIONNAIRE ON THE CORRELATION BETWEEN TYMPANIC MEMBRANE PERFORATION AND PATTERN OF HEARING LOSS.

SERIAL NO:

1. BIODATA
2. Initials:
3. Sex male female

3. Age: 15-24 25-34 35-44 4 45-54

55-64 65-74 75-84 ≥85

4. Marital status: Married

Single

Divorced

Widowed

5. Maximum level of education attained: None

Primary

Secondary

Tertiary

6. Occupation: Student

Self employed

Civil servant

Retired

Others (pls specify) ……………………..

1. HISTORY:

7. Duration of symptom days weeks month year

8. Symptoms (right ear) yes no (left ear) yes no

Otalgia

Otorrhoea

Impaired hearing

Ear itching

Tinnitus

Vertigo

Hemi facial weakness

Others (pls specify)………………………………………….

1. **PAST MEDICAL HISTORY** (right ear) yes no (left ear) yes no

Prior impaired hearing?

Ototoxicity from drugs/chemicals?

1. **CLINICAL EXAMINATION**

9. Signs (right ear) yes no (left ear) yes no

Otorrhoea

Tenderness

Cerumen auris

TM perforation

10. Position of perforation (Right or Left): Anteriosuperior

Posteriosuperior

Anterioinferior

Posterioinferior

Central

11. Size (area) of perforation (P)

12. Area of tympanic membrane (T)

13. Percentage area of perforation (P/T x 100%)

14. Audiometry

Hearing threshold (dB) right ear left ear
