# Supplementary material for: Correlating the site of tympanic membrane perforation with Hearing loss
Source: BMC Ear Nose Throat Disord. 2009 Jan 4;9:1. doi: 10.1186/1472-6815-9-1 (PMC2631525; doi:10.1186/1472-6815-9-1)
Supplement: Additional file 2 — bmc ent table 1.docx outlines the Frequency of Sites (locations) of tympanic membrane perforations. [file 1472-6815-9-1-S2.doc]

Table1: Frequency of Sites (locations) of tympanic membrane perforations.

| *Position* | *Left Side* | | *Right Side* | | *Total* | |
| --- | --- | --- | --- | --- | --- | --- |
|  | *Number of ears* | *%* | *Number of ears* | *%* | *Number of ears* | *%* |
| Central | 29 | 70.7 | 31 | 86.0 | 60 | 77.9 |
| Anteroinferior | 4 | 9.8 | 2 | 2.8 | 6 | 7.8 |
| Posteroinferior | 3 | 7.3 | 1 | 1.4 | 4 | 5.2 |
| Anterosuperior | 3 | 7.3 | 1 | 1.4 | 4 | 5.2 |
| Posterosuperior | 2 | 4.9 | 1 | 1.4 | 3 | 3.9 |
| Total | 41 | 100.0 | 36 | 100.0 | 77 | 100.0 |

Central perforation was the most frequent class of perforation encountered, whereas; posterosuperior is the least.
