# Supplementary material for: Correlating the site of tympanic membrane perforation with Hearing loss
Source: BMC Ear Nose Throat Disord. 2009 Jan 4;9:1. doi: 10.1186/1472-6815-9-1 (PMC2631525; doi:10.1186/1472-6815-9-1)
Supplement: Additional file 3 — bmc ent table 2a.docx illustrates the Statistical correlation of sites of perforations with hearing loss in acute TM perforations. [file 1472-6815-9-1-S3.doc]

Table 2 (a): Statistical correlation of sites of perforations with hearing     loss in acute TM perforations.

| *Site of perforation(left TM)* | *N* | *Mean hearing level*  *(dBHL)* | *SEM* |  |  |
| --- | --- | --- | --- | --- | --- |
| Central | 31 | 48.7 | 3.0 |  |  |
| Anterioinferior | 5 | 45.5 | 8.5 |  |  |
| Posteroinferior | 4 | 35.0 | 5.7 |  |  |
| Anterosuperior | 5 | 42.0 | 0.0 |  |  |
| Posterosuperior | 2 | 43.3 | 8.8 |  |  |
| Total | 47 | 46.27 | 3.5 |  |  |

| *K-W test* | **P** |
| --- | --- |
| 3.930 | 0.313 |
