# Supplementary material for: Correlating the site of tympanic membrane perforation with Hearing loss
Source: BMC Ear Nose Throat Disord. 2009 Jan 4;9:1. doi: 10.1186/1472-6815-9-1 (PMC2631525; doi:10.1186/1472-6815-9-1)
Supplement: Additional file 4 — bmc ent table 2b.doc shows Statistical correlation of sites of perforations with hearing loss in chronic TM perforations. [file 1472-6815-9-1-S4.doc]

**(b) Statistical correlation of sites of perforations with hearing loss in  chronic TM perforations.**

| *Site of perforation(right TM)* | *N* | *Mean hearing loss* | *SEM* |  |  |
| --- | --- | --- | --- | --- | --- |
| Central | 16 | 64.5 | 3.9 |  |  |
| Anterioinferior | 4 | 45.0 | 5.0 |  |  |
| Posteroinferior | 3 | 55.0 | 1.2 |  |  |
| Anterosuperior | 3 | 33 | 5.0 |  |  |
| Posterosuperior | 4 | 75 | 7.8 |  |  |
| Total | 30 | 59.18 | 3.8 |  |  |

| *K-W test* | **P** |
| --- | --- |
| 3.556 | 0.046 |

Where, TM- Tympanic membrane, SEM- Standard error of mean

K-W test– Kruskal-Walli’s testing

The sizes (% perforations=P/T x 100%) of perforation ranged from 1.51%-89.05%, with corresponding hearing levels 30dB – 90dB. The TM perforations showed a positive correlation with the magnitude of hearing loss (dBHL) using Pearson’s co relational test (p=0.01, r=0.05).

Tables 2a and 2b, show that 59% had pure conductive hearing loss and the rest were mixed type. The Sites\locations of perforations on the tympanic membrane were correlated with their Mean hearing levels (dB) using the Kruska-Walli’s Test (K-W Test). The coefficients of correlation (r) for ears with acute perforations (conductive) and chronic perforations (mixed) Tm perforations were 3.930 and 3.556, while their P- values (P) were 0.313 and 0.046 respectively. Tables 2 (a) and (b) **show** the sites of perforations with the Mean hearing losses, and the results of the statistical correlation.
