# Supplementary material for: Correlating the site of tympanic membrane perforation with Hearing loss
Source: BMC Ear Nose Throat Disord. 2009 Jan 4;9:1. doi: 10.1186/1472-6815-9-1 (PMC2631525; doi:10.1186/1472-6815-9-1)
Supplement: Additional file 5 — bmc ent table 2c.doc outlines Statistical correlation of sites of perforations with hearing loss in chronic TM perforations (mixed hearing loss) with elimination of the sensorineural component (A-B gap). [file 1472-6815-9-1-S5.doc]

Table 2(c) Statistical correlation of sites of perforations with hearing loss in chronic TM perforations (mixed hearing loss) with elimination of the sensorineural component (A-B gap).

| *Site of perforation(right TM)* | *N* | *Mean hearing loss*  *(Air-bone gap)* | *SEM* |  |  |
| --- | --- | --- | --- | --- | --- |
| Central | 16 | 41.0 | 3.2 |  |  |
| Anterioinferior | 4 | 22.0 | 4.7 |  |  |
| Posteroinferior | 3 | 35.0 | 2.1 |  |  |
| Anterosuperior | 3 | 13.0 | 3.0 |  |  |
| Posterosuperior | 4 | 55.0 | 6.2 |  |  |
| Total | 30 | 36.93 | 3.67 |  |  |

| *K-W test* | **P** |
| --- | --- |
| 2.214 | 0.049 |

Those perforations with minimal confounding factors tend not to correlate with site of perforations whereas, the long standing (chronic perforations) with associated confounding factors sensorineural hearing losses, possible ossicular erosions, cholesteatoma correlated with the site of perforations. worthy of note is that on elimination of the sensorineural component of the hearing loss (through the utilization of air-bone gap) , a similar result was obtained.
